# Associations among hair loss, oral sulfur-containing gases, and gastrointestinal and metabolic linked diseases in Japanese elderly men: pilot study

**DOI:** 10.1186/1471-2458-9-82

**Published:** 2009-03-13

**Authors:** Toshihiro Ansai, Shuji Awano, Inho Soh, Yutaka Takata, Akihiro Yoshida, Tomoko Hamasaki, Tadamichi Takehara

**Affiliations:** 1 Division of Community Oral Health Science, Department of Health Promotion, Kyushu Dental College, Kitakyushu, Japan; 2 Division of General Internal Medicine, Department of Health Promotion, Kyushu Dental College, Kitakyushu, Japan

## Abstract

**Background:**

Male pattern baldness (MPB), an observable trait, has been reported to be associated with various diseases, such as prostate cancer and cardiovascular disease. Oral sulfur-containing gases have also been suggested to be useful as markers of systemic health condition. However, there are no known reports regarding the associations among MPB, and oral sulfur-containing gases, and systemic health conditions in males.

**Methods:**

We studied 170 male subjects aged either 60 or 65 years old. The degree of MPB was assessed using the Norwood-Hamilton Baldness scale. Oral sulfur-containing gases were measured using a compact-designed device. All subjects completed physical and laboratory blood examinations, a face-to-face medical questionnaire, and an oral examination.

**Results:**

There were significant differences between the levels of CH_3_SCH_3 _and baldness patterns, independent of age. When we analyzed whether the association was linked to systemic health condition, a strong significant association was observed between the level of CH_3_SCH_3 _and severe MPB in subjects with gastrointestinal diseases, hypertension, and hypercholesterolemia.

**Conclusion:**

These results suggest that MPB is associated with the level of CH_3_SCH_3_, a sulfur-containing gas that causes oral malodor, in elderly Japanese males. Further, the association was intensified by the existence of gastrointestinal tract and metabolic disorders.

## Background

It has been reported that for diagnosis of systemic diseases, male pattern baldness (MPB), a clearly observable trait, can be used [1,2]. It was also noted that MPB appeared to be a risk factor for some diseases, for example, clinical prostate cancer and cardiovascular disease, independent of other risk factors, including race and age [3-5]. The precise mechanisms leading to the development of MPB and such related diseases are largely unknown, however, they share some epidemiologic and biological risk factors, including age, heritable genetic factors, and androgenic metabolism [6,7].

On the other hand, oral malodor is reported to be primarily associated with dental caries and periodontal disease, though involvement of other factors including systemic health conditions and lifestyle have not been ruled out. It is generally known that volatile sulfur-containing gases, which mainly consist of the three compounds hydrogen sulfide (H_2_S), methanethiol (CH_3_SH), and dimethylsulfide (CH_3_SCH_3_), are responsible for oral malodor [8]. However, it has not been clarified whether these sulfur gases are derived from the mouth and/or other organs such as the gut, or how the contents of these gases are involved with systemic health conditions. Sulfur-containing molecules in the oral cavity have been used as markers of various systemic conditions. For example, these sulfur gases are also present in colonic gas, though colonic concentrations are orders of magnitude greater than those in breath. Since these gases are rapidly absorbed from the gut, it is possible that they could be transported to the lungs via the bloodstream and then cleared in expired air, as is well documented to be the case with H_2 _and methane [9]. Colonic H_2_S and CH_3_SH in breath could be explained by an extremely efficient metabolism of these gases by the colonic mucosa or liver [10]. Further, another study suggested that subjects with chronic liver diseases could be differentiated from those with normal liver function by comparing the levels of some sulfur-containing compounds [11]. In contrast, CH3SCH3 is not metabolized by the colonic mucosa or the liver, and colonic CH_3_SCH_3 _might be expected to appear in breath, and Suarez et al. (2000)[9] suggested that some CH_3_SCH_3 _is derived from the gastrointestinal tract. In addition, Hoshi et al. (2002) [12] compared sulfur-containing gases between *Helicobacter pylori*-positive and negative patients, and found that the levels of H_2_S and CH_3_SCH_3 _in mouth air were significantly higher in positive patients. Considering that various systemic conditions affects breath in the mouth, as described above, it is possible that MPB has an association with levels of oral malodor.

The purpose of the present study was to investigate the severity of MPB in correlation with these sulfur-containing gases. We also investigated whether systemic health disorders were linked to changes in the levels of those gases.

## Methods

### Recruitment and data collection

This cross-sectional investigation was part of a population-based study conducted to investigate the relationships between oral and systemic health. A total of 170 healthy community-dwelling male subjects aged either 60 or 65 years old residing in Fukuoka Prefecture were recruited in 2006, and all were provided an explanation of the nature of the research project and provided written informed consent. The study was also approved by the Ethics Committee of Kyushu Dental College (no.05022250).

Each subject completed a questionnaire regarding lifestyle, oral and systemic health conditions, and medical history, and also underwent physical, laboratory blood, and oral examinations. All current medication usage was recorded, including treatments for hypertension, diabetes, and hyperlipidemia. Blood samples were taken after the subjects had refrained from oral intake and smoking for at least 2 h before collection, then transported to a commercial laboratory on ice for measurements.

The degree of MPB was assessed in the male subjects using Norwood-Hamilton Baldness scale [1] by a trained doctor, with hair loss graded progressively from Type I (no loss) to Type VII (hair loss complete at the crown). For the purpose of our analyses, subjects with MPB were then classified to three groups; slight (Type I and II), moderate (Type III including Type III vertex to V), and severe (Type VI and VII).

### Sampling of oral gases and the devise used

For sampling, subjects were asked to refrain from oral activity, including eating, drinking, tooth brushing, and mouthrinsing, before testing. Oral sulfur-containing gases were measured using an indium oxide (In_2_O_3_) semiconductor gas sensor (Oral Chroma, ABILIT, Tokyo, Japan), a compact-sized device that is popular in dental clinics in Japan, as described previously [13]. Briefly, subjects were instructed to keep their mouths closed and breathe through the nose for 30 s before analysis. A 1-mL disposable syringe was inserted into the oral cavity through the lips and teeth, and 1 mL of oral air was aspirated by the syringe. Immediately, 0.5 mL of the sample was injected into the device. In a previous hospital-based study that utilized the device (mean subject age, 32.1 years old), reference values for oral sulfur-containing gases were reported, as follows: H_2_S, 358.7 ppb (SE, 25.6); CH_3_SH, 143.0 ppb (SE, 14.6); CH_3_SCH_3_, 19.5 ppb (SE, 2.3) [14].

### Statistical analysis

To assess differences between groups, a chi-square test was used for categorical variables and ANOVA for continuous variables, followed by adjustment for the effects of age if necessary. Linear trends for the associations were assessed by computing the *P *value for each trend with multiple regression analysis. All statistical analyses were performed using SPSS 14.0 for Windows (SPSS Japan, Tokyo, Japan). The level of statistical significance was set at 0.05 for all of the analyses.

## Results

The prevalence and distribution of MPB in the 170 male subjects is shown in Table 1. We classified 67 subjects as slight (39.4%), 64 as moderate (37.6%), and 39 were severe (23.0%). As shown in Table 2, there were significant differences among the groups in regard to age, but not regarding systemic conditions, including blood pressure and laboratory blood test results, or lifestyle factors of alcohol use and smoking habit. Medical history for the subjects revealed 68 with hypertension (of whom 46 were receiving medication), 25 with diabetes, 10 with CVD, 24 with CHD, 39 with hepatic disease, 15 with respiratory disease, 79 with gastrointestinal disease, and 26 with hypercholesterolemia. There were no significant differences among the groups in regard to medical history. Table 3 shows the oral health status separated by MPB group. There was a significant difference only for the level of CH_3_SCH_3_. There were no significant differences between the ages groups regarding oral sulfur-containing gases. The mean values for H_2_S, CH_3_SH, and CH_3_SCH_3 _for all subjects were 4.64 ng/10 mL (= 346.6 ppb), 2.82 ng/10 mL (= 149.5 ppb), and 0.54 ng/10 mL (= 22.2 ppb), respectively. We also investigated the effects of systematic health on CH_3_SCH_3 _levels, as shown in Table 4. In the subjects with gastrointestinal diseases, hypertension (treated), and hypercholesterolemia, there were significant differences among the groups. In particular, CH_3_SCH_3 _in those with severe MPB showed significant associations with gastrointestinal disease, hypercholesterolemia, and hypertension (treated) (*P *for trend < 0.05 for each), with gastrointestinal disease and hypercholesterolemia occurrences shown to be approximately 2 to 5-fold higher than the level shown in Table 3. A total of 79 subjects had gastrointestinal diseases, which were gastritis in 24, gastric ulcers in 12, duodenal ulcers in 10, gastric cancer in 1, large intestine polyps in 11, and combinations of those gastrointestinal diseases in 21. In contrast, there were no significant differences among the groups in regard to subjects treated to eliminate *H. pylori*. In addition, in subjects with other systemic diseases such as hepatic and diabetic diseases, there were no significant differences among the groups regarding CH_3_SCH_3 _levels.

**Table 1:** Distribution of the baldness patterns in male subjects

Baldness type	N (%)
I	35 (20.6)
II	32 (18.8)
III	26 (15.3)
IV	25 (14.7)
V	13 (7.6)
VI	18 (10.6)
VII	21 (12.4)

**Table 2:** General characteristics of subjects divided by baldness group

Characteristic	Slight	Moderate	Severe	P*
Number of subjects	67	64	39	
Age (years)	61.0 (2.1)	61.6 (2.3)	62.7 (2.5)	0.002
Height (cm)	165.9 (5.7)	165.2 (6.5)	163.4 (4.9)	0.11
Weight (kg)	68.0 (9.9)	65.6 (9.7)	64.2 (7.9)	0.10
Body mass index	24.7 (3.1)	23.9 (3.0)	23.9 (2.5)	0.33
Systolic blood pressure (mmHg)	146.2 (16.9)	146.9 (21.6)	144.6 (22.2)	0.86
Diastolic blood pressure (mmHg)	84.3 (10.1)	83.5 (10.0)	84.4 (12.2)	0.88
Serum total cholesterol (mg/dL)	208.3 (34.7)	203.9 (32.9)	208.2 (30.5)	0.72
HDL cholesterol (mg/dL)	57.8 (15.0)	59.5 (16.1)	59.3 (14.2)	0.79
HbA1c (%)	5.30 (0.71)	5.27 (0.73)	5.33 (0.96)	0.93
Estradiol (E2) (pg/mL)	25.7 (6.4)	25.4 (6.1)	25.9 (6.4)	0.92
Testosterone (pg/mL)	13.2 (4.5)	12.7 (3.7)	13.2 (3.9)	0.76
Smoking habit				
Never	13	17	13	0.47
Past	36	35	17	
Current	18	12	9	
Alcohol use				
Never	3	7	3	0.66
Light	9	7	4	
Moderate	19	13	13	
Severe	36	37	19	
Medical history				
Hypertension	27	27	14	0.67
Diabetes	11	9	5	0.96
CVD	3	5	2	0.70
CHD	6	10	8	0.36
Hepatic	14	15	10	0.83
Respiratory	7	7	1	0.41
Gastric	28	31	20	0.57
Hypercholesterolemia	15	8	3	0.098

Values indicate number or mean (SD).

CVD: cardiovascular disease; CHD: coronary heart disease.

*Differences between groups were assessed using a chi-square test for categorical variables and an ANOVA for continuous variables.

**Table 3:** Oral health status of subjects divided by baldness group

Parameter	Slight	Moderate	Severe	P**
Number of carious teeth	0.43 (0.9)	0.67 (1.7)	0.69 (1.3)	0.49
Number of missing teeth	5.82 (5.7)	4.19 (4.7)	6.21 (6.7)	0.13
Maximum PD (mm)	4.03 (1.4)	4.17 (1.7)	4.59 (1.5)	0.19
Maximum CAL (mm)	5.32 (1.9)	5.31 (2.3)	5.28 (2.9)	0.99
Number of BOP	3.08 (3.4)	2.86 (3.8)	3.33 (4.3)	0.82
Stimulated salivary flow (mL/min)	1.30 (0.88)	1.37 (0.77)	1.36 (0.66)	0.87
H_2_S (ng/10 mL)	4.34 (6.7)	4.74 (6.9)	4.99 (7.0)	0.89
CH_3_SH (ng/10 mL)	2.29 (4.7)	2.56 (4.4)	4.13 (7.3)	0.21
(CH_3_)_2_S (ng/10 mL)	0.33 (0.7)	0.27 (0.5)	1.31 (0.3)	0.005

Values indicate mean (SD).

PD: probing depth; CAL: clinical attachment loss; BOP: bleeding on probing.

*Differences between groups were assessed using an ANOVA.

**Table 4:** Mean values of (CH_3_)_2_S in subjects with systemic disease divided by baldness group

	Slight	Moderate	Severe	P*	P**
Gastrointestinal disease (N = 79)	0.31 (0.80)	0.19 (0.39)	2.26 (4.38)	0.001	0.009
Hypertension (N = 68)	0.47 (0.94)	0.21 (0.35)	0.96 (1.36)	0.02	0.22
Hypertension (treated) (N = 46)	0.27 (0.65)	0.25 (0.39)	1.30 (1.66)	0.007	0.02
Hypercholesterolemia (N = 24)	0.13 (0.36)	0.16 (0.28)	6.50 (11.09)	0.009	0.04
*H. pylori *elimination (N = 13)	0.45 (0.77)	0.05 (0.08)	0.24 (0.39)	0.43	0.61
Hepatic disease (N = 39)	0.46 (0.80)	0.18 (0.31)	0.59 (1.26)	0.31	0.79
Diabetic disease (N = 25)	0.43 (0.94)	0.12 (0.21)	1.61 (2.91)	0.18	0.24

Values indicate mean (SD) (ng/10 mL).

*P value assessed by ANOVA.

**P value for trend assessed by multiple regression analysis.

## Discussion

In the present cross-sectional study, we investigated community-dwelling elderly subjects and found a significant association between CH_3_SCH_3 _levels in volatile sulfur-containing gases and MPB. Further, and the level of CH_3_SCH_3 _was found to be much greater in males with gastrointestinal diseases, hypertension, and hypercholesterolemia. In general, oral sulfur-containing gases have been reported to be influenced by systemic conditions [15], though the number of epidemiological investigations is limited. A recent study suggested that CH_3_SCH_3 _is the main contributor to extra-oral or blood-borne halitosis, due to a hitherto unknown metabolic disorder, while CH_3_SH and H_2_S are the main contributors to intra-oral halitosis [16]. A non-oral etiology of halitosis may include disturbances of the upper and lower respiratory tracts, disorders of the gastrointestinal tract, metabolic disorders, and carcinomas [17]. The review has also suggested that halitosis originating from the respiratory tract is due to chronic sinusitis, chronic tonsilloliths, or nasal obstruction, while halitosis originating from the gastrointestinal tract is due to inflammatory bowel disease, *H. pylori *infection, gastritis, and esophageal reflux disease [17]. Most recently, Zhang et al. suggested that gastrointestinal symptoms including halitosis, gastric reflux and abdominal bloating, is significantly associated with sebaceous gland diseases (seborrhea, seborrheic dermatitis, acne, androgenetic alopecia and rosacea) [18], though their study was based on the questionnaire investigation. In the present study, we found it interesting that there was no significant association regarding oral sulfur-containing gases in our subjects when divided into those with and without gastrointestinal diseases (data not shown). Nevertheless, higher levels of CH_3_SCH_3 _were observed in those with gastrointestinal diseases. Considering the correlations among halitosis, gastrointestinal diseases, and sebaceous gland diseases as described above, it is reasonable that a combined effect of MPB and gastrointestinal disease may lead to increased CH_3_SCH_3_.

Hoshi et al. (2002) [12] compared the levels of oral malodor between *H. pylori*-positive and -negative patients, and reported that levels of H_2_S and CH_3_SCH_3 _in mouth air were significantly higher in the positive patients. Our results also showed that levels of CH_3_SCH_3 _were lower in subjects who had received treatment to eliminate *H. pylori*, though the difference was below the level of significance. On the other hand, the levels of H_2_S were scarcely changed. Considering that H_2_S originates from microbial putrefaction within the oral cavity [16], this finding may be partly explained by the effect of elimination of *H. pylori*, which might be no greater than that of CH_3_SCH_3_. An intervention study is needed to clarify this relationship.

On the other hand, in subjects with hypertension or high cholesterol, stronger associations between MPB and CH_3_SCH_3 _levels were observed. There are no known reports regarding associations between halitosis and systemic diseases, such as hypertension and hypercholesterolemia, whereas several studies have indicated that MPB may be a risk factor for some diseases, such as CVD or prostate cancer, though the detailed mechanisms are unknown [3-5]. One plausible mechanism has been speculated to be related to sex hormones, such as androgens [3]. The principal androgen responsible for MPB may be dihydrotestosterone (DHT), since it is an active metabolite of testosterone produced in tissue by the action of 5α-reductase, and is involved in the pathogeneses of both MPB and myocardial infarction [19]. A recent investigation showed that the level of DHT and ratio of testosterone to epitestosterone in vertex hair from premature baldness subjects were higher than in the sample of non-baldness subjects [20]. However, we could not clarify the associations between MPB and levels of sex hormones in the present study, since the level of DHT was not measured in our subjects.

Another recent report [21] found an association between BMI and oral malodor, and suggested that alcohol intake and BMI may be factors to help predict oral malodor. High BMI has also been associated with a variety of ailments, including type II diabetes, hypertension, dyslipidemia, cerebrovascular accident, myocardial infarction, cancer (e.g., prostate cancer and colon cancer), gout, arthritis, fatty liver, and sleep apnea [22]. However, we found no significant differences among the groups in regard to height, weight, and BMI. Considering these findings, the association between MPB and oral malodor may be explained through novel mechanisms that have not been defined.

There are some limitations to our study. First, the number of subjects was limited and the results were obtained in a preliminary research manner. Further, the study subjects were generally in good health, thus our findings may indicate that an association that exists primarily in healthy elderly subjects. Finally, because all subjects were Japanese, thus our results may not be applicable to men of other racial groups.

## Conclusion

From this study, within the limitations of the present study, our results suggest that MPB is associated with an increased risk of oral malodor independent of age, while the existence of gastrointestinal disease, and known risk factors for CVD and CHD (i.e., hypertension and hypercholesterolemia) were found to strengthen association between MPB and CH_3_SCH_3 _levels, though the causes and effects remain unclarified. Conversely, it is possible that a subject with higher levels of CH_3_SCH_3 _and severe MPB may be predisposed to gastrointestinal diseases and cardiovascular-related diseases. If so, then assessment of MPB and measurement of oral malodor, simple and non-invasive methods, may be useful as screening for potential systemic health conditions, including disorders in the gastrointestinal tract and metabolic disorder, in a large population-based examination. Nevertheless, additional research is needed to corroborate these findings and clarify the biological mechanisms related to the increase in CH_3_SCH_3 _levels in subjects with severe MPB.

## Competing interests

The authors declare that they have no competing interests.

## Authors' contributions

The epidemiological study was supervised by TT and TA. TA, SA, IS, YT, AY, TH, TT, participated in the epidemiological study. TT initially designed the study. TA contributed towards analysis and interpretation of data, and wrote the first draft of the manuscript. All authors read and approved the final version of the report.

## Pre-publication history

The pre-publication history for this paper can be accessed here:


